# Prevalence and associated factors of gastrointestinal helminthiasis of lactating cow and effect of strategic deworming on milk quantity, fat, and protein in Kucha, Ethiopia

**DOI:** 10.1186/s12917-022-03251-2

**Published:** 2022-04-25

**Authors:** Fikre Haymanot, Tamirat Kaba

**Affiliations:** 1 Kucha District Agricultural Bureau, Livestock and Fisheries Resource Unit, Kucha, Ethiopia; 2 Department of Veterinary and Animal Science, College of Agricultural Science, Arba Minch University, P.O. Box: 21, Arba Minch, Ethiopia

**Keywords:** Gastrointestinal helminth, Kucha, Milk fat, Milk protein, Milk yield, Prevalence

## Abstract

**Background:**

Gastrointestinal helminthiasis poses economic impacts on the dairy sector by reducing milk production. This study aimed at estimating the prevalence of gastrointestinal helminthiasis, the burden of helminths, and appraising potential factors associated with the prevalence in lactating cows. The study was also designed to elucidate the effects of anthelmintic treatment on milk yield, milk fat, and protein content in the Kucha district.

**Methods:**

A cross-sectional and field clinical trial study designs were used. Standard parasitological techniques (floatation and sedimentation) were employed to detect cows’ infection status. McMaster and Stoll’s egg counting methods were used to estimate helminths' burden. All putative factors that might have been linked with infection were recorded by field observations and farmers' interviews. Sixty cows tested positive for the parasitic infection in the cross-sectional study design were randomly assigned into one of the two groups (dewormed Vs control). Milk yield, milk fat and protein contents were recorded in both groups on day zero and then on weekly basis. Descriptive statistics, binary logistic regression, and repeated measure ANOVA were used to analyze the data.

**Results:**

Overall, of 422 examined cows, 150 (35.5%, 95% CI; 30.9-40.3%) were infected with at least one of the gastrointestinal helminth parasites. Strongyle nematode was the predominant type accounting for 52% of the total record. Analysis of fecal egg count (FEC) in infected cows depicts the highest record of *Parampistomum* egg, accounting 457.14 ± 275.45 Egg per gram (EPG) of feces. The prevalence of gastrointestinal helminthiasis in cow that had an average body condition score of 1.7 was over two fold higher (OR = 2.19, 95% CI = 1.17–4.17, *P* = 0.016) than in cows with 6.3 body condition score. A significant improvement in milk yield, milk fat, and milk protein was observed in dewormed cows over 28 days period.

**Conclusion:**

Gastrointestinal helminthiasis is threatening the welfare of lactating cows in Kucha, Ethiopia given its negative association with the body condition score. Gastrointestinal helminths are responsible for the reduction of milk yield and loss of milk fat and protein. However, an improvement in milk yield, milk fat, and milk protein after deworming using Tetrox® (Tetramisole and Oxyclozanide combination), a new drug to the area/ a drug used by relatively few farmers in the study area, proves the effectiveness of strategic deworming.

**Supplementary Information:**

The online version contains supplementary material available at 10.1186/s12917-022-03251-2.

## Background

The global demand for cow's milk is on the rise. This results from poor production and/or possibly from the ever-increasing world population. Although progressive improvement has been seen in the developing world as regards the production of milk and its products [1] the demand remains much higher than supply. This current imbalance between production and human demand for dairy products is an opportunity to invest in the dairy sector in the future. However, various productions related technical constraints that have been affecting the dairy sector in past years are expected to continue being major threats to the sector. Of several constraints, parasitic diseases in general, and gastrointestinal helminthiasis in particular, are thought to be the major challenges [2].

Gastrointestinal helminthiasis is a parasitic infection caused by a group of helminth parasites, which affect the gastrointestinal tract (GIT), associated organs, and whose eggs are excreted to the environment through animals’ faecal material. This group of parasites comprises a variety of species of roundworms, including genus *Haemonchus, Trichostrongylus, Trichuris, Bunostomum, Oesophagostomum, Toxocara vitulorum* (*T. vitulorum*), and others. Gastrointestinal helminth also comprises flatworms, such as *Fasciola, Paramphistome*, and others flukes [3].

Many researchers across the globe have reported a wide range of prevalence for specific gastrointestinal helminth parasites in dairy cows [7–11]. The prevalence variations of specific gastrointestinal helminth across different regions suggest the influence of several epidemiological factors on the magnitude of the infection. Of many epidemiological factors, husbandry management in general, and dewormingin particular, using available anthelmintic drugs appears to be the major strategy to lessen the impacts of gastrointestinal helminthiasis infection in food-producing animals. Various anthelmintics can successfully eliminate the parasites; despite resistance development is challenging the intended outcomes [12].

In Kucha, Ethiopia, dairy products, such as milk and butter are considered the major resources base. “*Ye Kucha Kibe*" literally meaning "butter from Kucha" is a name known by the majority of Ethiopians to indicate "high-quality butter". However, a study shows diseases affect butter production in the district [13], despite this study does not demonstrate the type of disease. Although gastrointestinal helminthiasis is considered a major problem of the food-producing ruminants in the country, many studies have focused the condition on the small ruminants, and meanwhile there is a paucity of information on the epidemiology of the condition at the country level in general, and in the Kucha district in particular. In addition to various factors, husbandry managements, such as deworming using anthelmintic compound has become the most widely practiced control method. However, a tendency to use chemically dissimilar anthelmintic drugs for a longer period and their utilization in an irrational way by the smallholder farmers often pose a risk of anthelmintic resistance [14]. Gastrointestinal helminthiasis management in such a way does not appear to eliminate parasite burden, and hence does not bring production improvement in lactating cows. Thus, we hypothesized that deworming using a new anthelmintic compound or anthelmintic drug seldom utilized by the farmers in the district would improve milk quantity and quality, when there is no significant difference in the magnitudes of gastrointestinal helminthiasis between dewormed and non-dewormed groups.

Therefore, the objective of this study was to estimate the prevalence, appraise associated factors of gastrointestinal helminthiasis and to elucidate the effect of anthelmintic treatment on milk quantity, fat, and protein contents in lactating cows in Kucha, Ethiopia.

## Results

### Husbandry practices

Data on husbandry practices of dairy cow were collected from 422 farmers, which prove 100% response rate. Accordingly, 49% of farmers showed their herd size to be 1–3 dairy cows. The majority of respondents indicated the presence of only one lactating cow (64.2%) with no other domestic animals at home (83%). Over 95% of lactating cow were local Zebu, of which almost 95% being managed extensively. The majority of the cow were young, having an average condition score of 2.6 and at mid-lactation period. The highest proportion of farmers highlighted that they use Albendazole bolus (65.4%), followed by Tetramisole, Ivermectin, and Tetrox (combination of Tetramisole and Oxyclozanide) to lessen the impacts of gastrointestinal helminthiasis (Table 1).

**Table 1:** Informants’ response (*N* = 422) on overall husbandry practice of dairy cow production in Kucha, Ethiopia

Variable	Number	Percent
**Dairy cow herd size**		
1–3 animal	206	49
4–6 animal	180	42
> 6 animal	36	9
**No. of the lactating cow**		
1 cow	271	64.2
2 cows	108	25.6
3 cows	43	10.2
**Breed**		
Zebu	404	95.7
Zebu x HF cross	18	4.3
**Age**		
young (4–7 years)	289	68.5
adult (8–10 years)	16	3.8
old (> 10 years)	117	27.7
**Lactation stage**		
early (calving-3 months)	145	34.4
mid (3–6 months)	161	38.2
late (> 6 months)	116	27.4
**Average body condition score**		
1.7	87	20.6
2.6	252	59.7
6.3	83	19.7
**Presence of other species of animals**		
yes	72	17
no	350	83
**Type of anthelmintic used**		
Albendazole bolus	276	65.4
Ivermectin	35	8.3
Tetramisole	86	20.37
Tetrox	25	5.93
**Management**		
extensive	400	94.7
semi-intensive	22	5.3

*HF* Holstein Frisian

### Gastrointestinal helminthiasis prevalence

Overall, of 422 examined cows, 150 (35.5%, 95% CI; 30.9–40.3) were infected with at least one type of gastrointestinal helminth parasites. The highest prevalence was observed in the multiparous (37.5%); old (43.75%), cow with 1.7 body condition score (47.12%) and cow at late lactation stage (40.51%) that lives in the highland agro-ecology (50%). In addition, slight variation was noted in the prevalence of gastrointestinal helminthiasis in the categories of various explanatory variables, including breed, deworming history, management system and pregnancy status (Additional file 1).

Of infected cows (*n* = 150), the majority (68%) had a mono-parasitic infection while only 32% of cows had a poly-parasitic infection. Strongyle was the predominant nematode among mono-infection accounting 52%, followed by *Fasciola* (11.3%) and *Paramphistomum* (4.7%)*.* Of the poly-parasitic infected cows, 26.7% had two parasite combinations, particularly Strongyle and *Paramphistomum* spp*.* mixture being the dominant group. *Moniezia, Trichuris* and *Ascarid* spp*.* often appeared in mixed form with others or each other, whereas Strongyle nematode detected in all mono- and poly-infections (Table 2).

**Table 2:** Prevalence of mono and poly-parasitic infections in lactating dairy cow in Kucha district

**Poly infections**	No. positive (%)	**Mono- infection**	No. positive (%)
***Two helminth parasites***			
Strongyle + *Fasciola* spp.	14 (9.3)	Strongyle	78 (52)
Strongyle + *Moniezia* spp.	7 (4.7)	*Fasciola* spp.	17 (11.3)
Strongyle + *Paramphistomum* spp.	16 (10.7)	*Paramphistomum spp.*	7 (4.7)
Strongyle + *Trichuris* spp.	3 (2)		
Subtotal	40 (26.7)		
***Three helminth parasite***			
Strongyle + *Fasciola* + *Moniezia* spp.	2 (1.3)		
Strongyle + *Ascarid* + *Paramphistomum* spp.	2 (1.3)		
Strongyle + *Trichuris* + *Paramphistomum* spp.	2 (1.3)		
Strongyle + *Ascarid* + *Moniezia* spp.	2 (1.3)		
Subtotal	8 (5.3)		
**Overall**	**48 (32)**	**Overall**	102(**68**)

### Burden of gastrointestinal helminths of the lactating cow

**Fig. 1:**
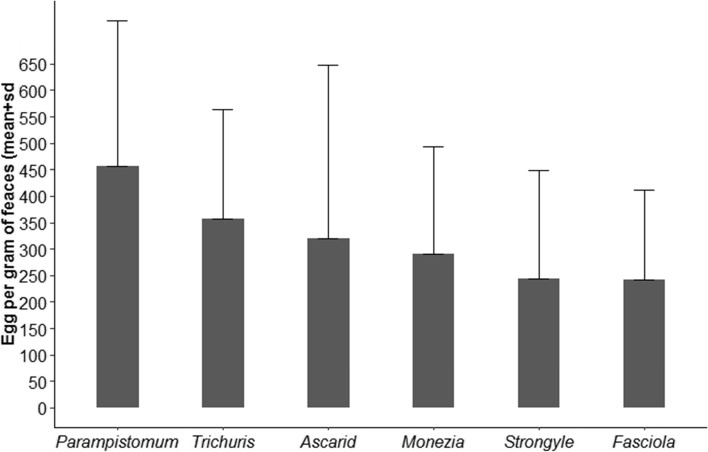
Mean egg per gram of feces of helminth parasites of lactating cow in Kucha district, Ethiopia

Analysis of fecal egg count (FEC) in infected cows depicts the highest (mean ± sd) record of *Parampistomum* egg, accounting 457.14 ± 275.45 Egg per gram (EPG) of faeces, followed by *Trichuris* (357.14 ± 207.02), *Ascarid* (320 ± 327.11), *Moniezia* (290.91 ± 206.26), Strongyle (242.86 ± 205.01) and *Fasciola* (242.42 ± 169.61) (Fig. 1).

### Risk factor analysis

Several host and husbandry factors' relationship with the prevalence of gastrointestinal helminthiasis is given in Additional file 1. Logistic regression analysis reveals a significant negative association of gastrointestinal helminthiasis and body condition of cow. Accordingly, the prevalence of gastrointestinal helminthiasis in cow with 1.7 condition score was over two fold higher (OR = 2.19, 95% CI = 1.17–4.17, *P* = 0.016) than in cows with 6.3 body condition score. Moreover, the prevalence of gastrointestinal helminthiasis in cows, which had 2.6 body condition score was insignificantly higher by 25% compared to the reference group (OR = 1.25, 95% CI = 0.73–2.18, *P* = 0.485). Meanwhile, there was no adequate evidence of association of infection with other hypothesized factors, such as agro-ecology, age, breed, pregnancy status, deworming history, lactation period, parity and management system.

### Effect of deworming on milk quantity

**Fig. 2:**
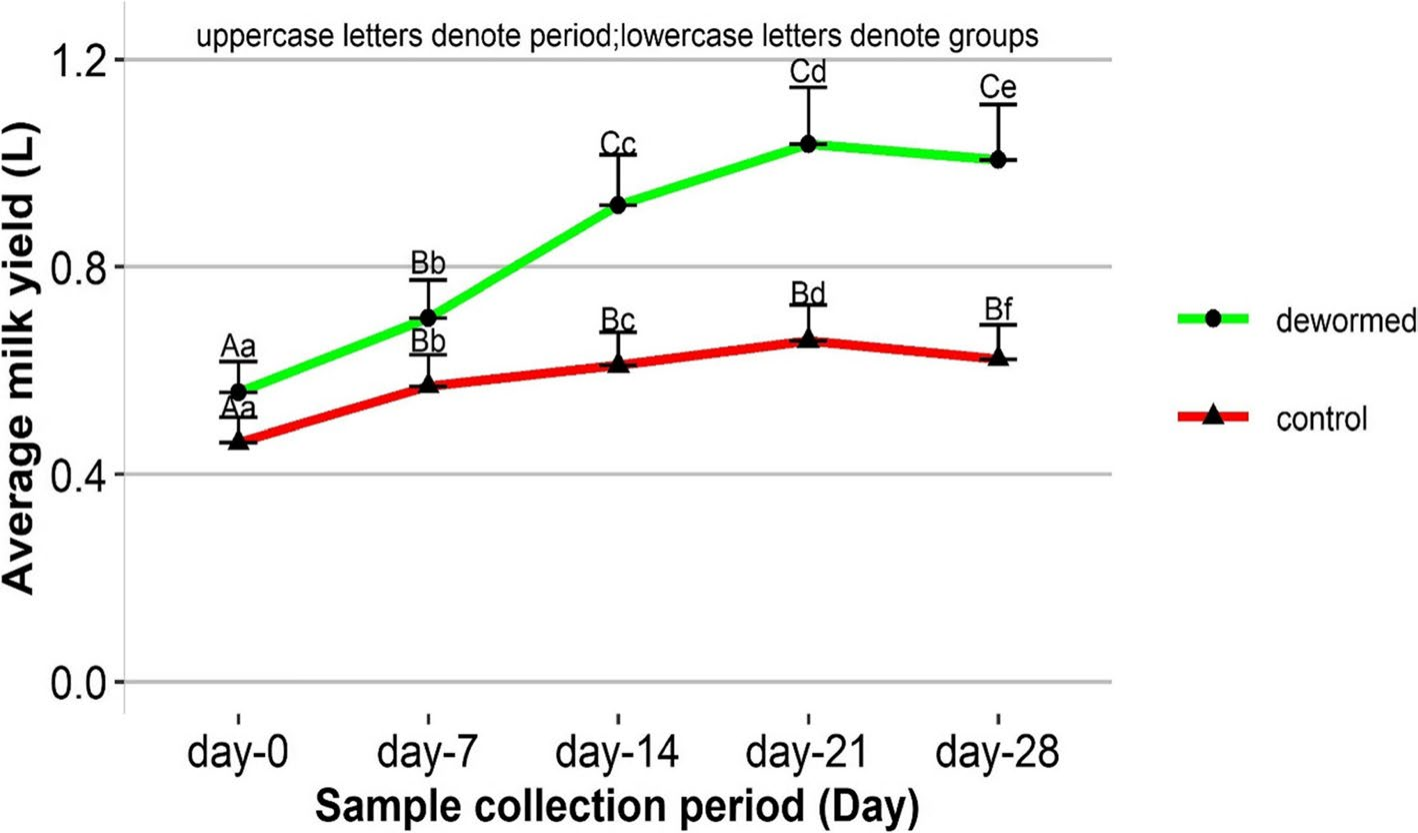
Milk yield in dewormed and control cows over 28 days follow up period. Values are presented as LSM + SE. Different uppercase letters within a group indicate significant between the period while different lowercase letters depict significant between the groups within a follow up period at *P* < 0.05

A two-way repeated measure ANOVA indicates a statistically significant (*P* < 0.05) effect of the period (days) and the interaction of treatment and period (treatment*period) on log (x-5). A back-transformed average milk values of dewormed (0.558 L) and control (0.461 L) groups of the cow did not significantly vary (*P* = 0.9551) on the initial day (D-0) of the trial. Following the initial day (D-0), an average milk quantity steadily climbed in both dewormed and control cows with significant variations (*P* < 0.05) from week to week until the value hit the peak by 21 days followup time. Average milk value slightly declined without significant change (*P* > 0.05) thereafter in both groups and remained over 1 L in dewormed cows and under a liter in the control group by the end of the study. The average milk amount of both dewormed and control groups by the end of the study showed a statistically significant gain (+ 0.449 L) and (+ 0.161 L) respectively compared to their initial values (*P* < 0.001). However, these gains in dewormed (+ 0.449 L) and control (+ 0.161 L) cohorts of the cow are statistically significant (*P* = 0.049) (Fig. 2).

### Effect of deworming on milk fat

**Fig. 3:**
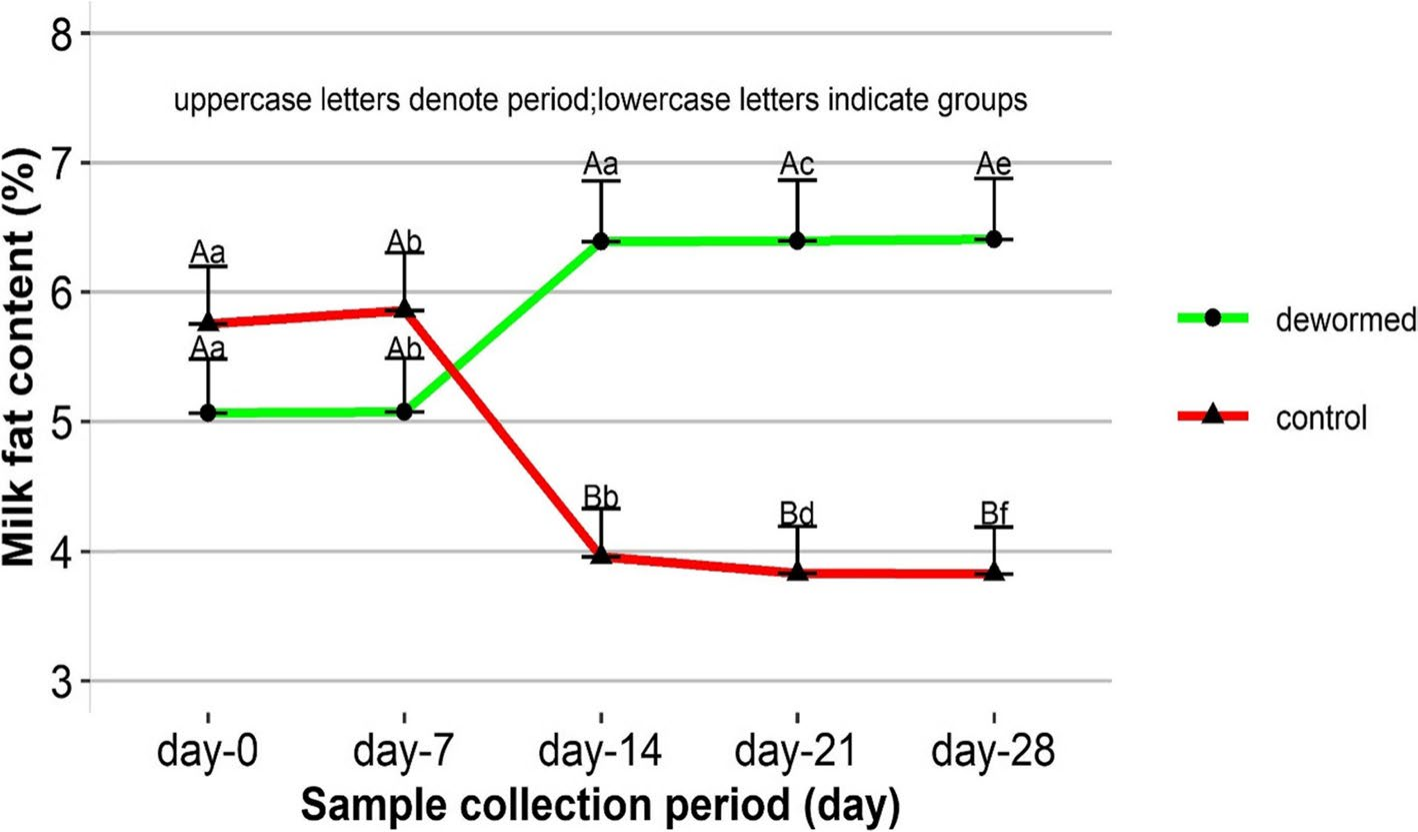
Milk fat content in dewormed and control cows over 28 days follow up period. Values are presented as LSM + SE. Different uppercase letters within a group indicate significant between the period while different lowercase letters depict significant between the groups within a follow up period at *P* < 0.05

The analysis revealed a statistically significant (*P* < 0.05) effect of period (days) and the interaction of treatment and period (treatment*period) on the square root (x + 5). The initial values of fat content in dewormed (5.06%) and control (5.75%) cow did not significantly vary (*P* = 0.9809). The fat content of the final day (day-28) in dewormed and control groups was 6.41% and 3.83% respectively. The difference of initial and final values in dewormed group shows an increment (+ 1.35%) however, this gain is not significant (*P* = 0.126). On the other hand, this difference reveals progressive significant (*P* < 0.001) loss (-1.92%) in the control group (Fig. 3).

### Effect of deworming on milk protein

**Fig. 4:**
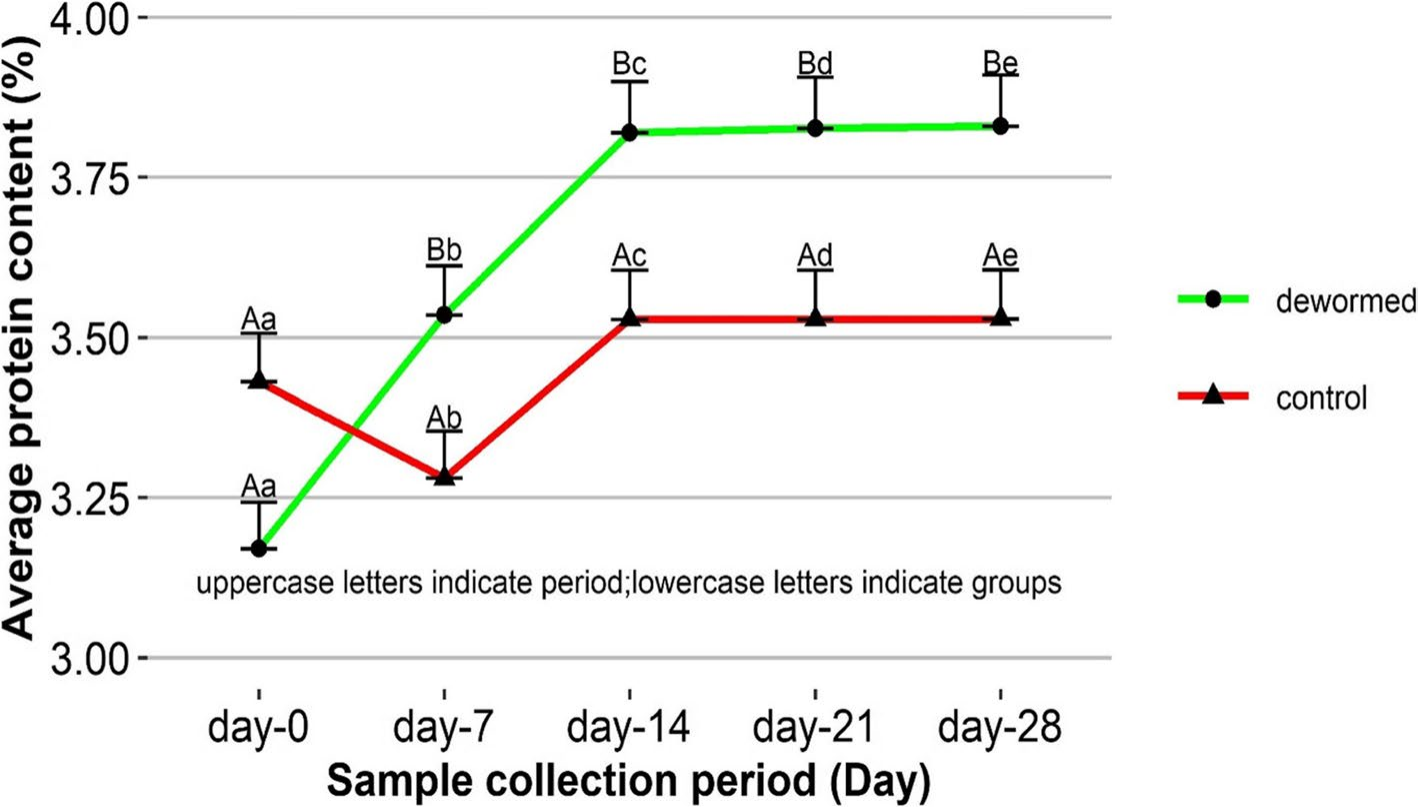
control cows over 28 days follow up period. Values are presented as LSM + SE. Different uppercase letters within a group indicate significant between the period while different lowercase letters depict significant between the groups within a follow up period at *P* < 0.05

A two-way repeated measure ANOVA revealed statistical associations of treatment, period, and their interaction (*P* < 0.05). Accordingly, initial values of milk protein of dewormed (3.17%) and control (3.43%) cows were not significantly different (*P* = 0.2858). The initial value and the value at the end of the study (3.83%) were statistically significant (*P* < 0.001) in dewormed animals, however, these values (3.43 Vs 3.53%) did not significantly vary (*P* = 0.9896) in control cow (Fig. 4).

## Discussion

This study aimed at estimating the prevalence of gastrointestinal infection of lactating cows and appraising associated factors in Kucha districts, Ethiopia. It also aimed at elucidating the effect of deworming with a new (not being frequently utilized by household) anthelmintic drug on the milk yield, milk fat, and protein contents.

### Husbandry practices

In the Kucha district, dairy production is one of the methods to generate household income. However, the production system seems suboptimum as many households often keep few number of dairy cows (Table 1). This observation is in line with the study from Jimma, Ethiopia [15] that demonstrated an average herd size of five dairy animals. The possible reason for keeping a few herds in the current study could be associated with inadequate grazing land. This explanation can also be viewed from the point of absence of other domestic animals for the majority of dairy producers in the area (Table 1). The presence of only one lactating cow at home, for the majority of households in the present study also agrees with the report of Fikru (2012) [13]. A tendency to use Albendazole bolus by the majority of households in the district concords with a study from Bedele, Ethiopia [16].

### Prevalence and burden of gastrointestinal helminths in lactating cow

The overall prevalence of gastrointestinal helminthiasis of lactating cows in the current study could not easily be compared with other studies conducted in either Ethiopia or elsewhere as our methodology was different from many other studies (use of only lactating cow versus general cattle/dairy cattle). Nonetheless, we tried to the level we could in order to bring existing literature for the comparisons with our results. In this regards, an overall prevalence in our study corresponds with the prevalence report from Holleta, Ethiopia [17]. Meanwhile, our prevalence estimate was lower than the prevalence recorded in other districts of Ethiopia [18, 19], and higher when compared with a similar study in other country (Nepal), in which only lactating cows were participated [20]. This variation could be due to the difference in husbandry practices, host susceptibility, agro-ecology, and methodology. The highest prevalence estimate of strongyle nematode among identified helminths in the present study suggests its significance in lactating cows. Similar findings have been reported in Jimma, Ethiopia [19] and India [21]. Contrarily, the aforementioned study in Nepal [20] shows that *Fasciola* was the most dominant parasite of lactating cows. This difference might be attributed to the variations in the agro-ecology of the region, in which the effect of climatic factors dictate the biology of individual helminth parasites [22].

The faecal egg estimates for all helminths identified in the current study indicate a mild form of infection [3]. However, given the 32% prevalence of mixed infection (Table 2), the observed burden for each helminth should not be overlooked. The highest fluke EPG of faeces for *Paramphistomum,* which was relatively less prevalent helminth, might suggest the development of resistance against anthelmintic drugs being used in the area. On the other hand, relatively the lower faecal egg count of the most dominant gastrointestinal helminths (strongyle) is interesting. The observed lower egg counts could be due to the presence of a few anthelmintic resistant strongyles, which have a low fecundity profile.

### Risk factors

The risk factors investigation reveals there was not adequate evidence for almost all hypothesized factors to be significantly associated with gastrointestinal helminthiasis in lactating cow except, body condition score. This implies gastrointestinal helminths infection is equally important in lactating cows irrespective of their difference in host factors, production stages, and husbandry methods. As expected, the significant associations of cows' body condition score and the prevalence of gastrointestinal helminthiasis depict the importance of gastrointestinal helminths in the welfare of dairy cows. The effect of helminthiasis on general conditions of cows possibly due to progressive reduction in body weight [23] or/and the inability of poorly conditioned cows to completely reject worms or prevent larva establishment due to weak immunity as ascribed elsewhere [24]. Moreover, a non-significant difference of helminths infection prevalence in a dewormed and non-dewormed group of cows indicates a therapeutic failure, possibly due to the emergence of anthelmintic resistance by the parasites. Although therapeutic failure for a drug, Albendazole has not yet been documented in cattle in Ethiopia; its poor quality in terms of active pharmaceutical ingredients [25] and its utilization by the majority of farmers in the current study might have contributed to the therapeutic failure.

### Effect of deworming on milk yield, fat and protein content

Significant variations of milk yield between the dewormed and control cow at the final study period (day-28) suggests an improvement of milk yield after intervention against gastrointestinal helminthiasis. Elimination of adult helminths burden by the anthelmintic drug might have reduced stress caused by the parasitism, which contributed to the rise of milk quantity. This finding concords with the report from Nepal [20], Wales [26], and Austria [27] that prove a significant improvement in milk yield after deworming. In the meantime, a progressive increment of milk yield over a period in control cows might be attributed to the inclusion of cows at early and mid-lactation stages in which a gradual rise of milk yield over a certain specific period could have taken place [28].

An improvement of milk fat in dewormed cows whilst a progressive loss of fat content in control cow points to the negative impact of gastrointestinal helminths in the milk fat content. The finding in the Nepal [20] that illustrates an improvement of milk fat content in the dewormed and its reduction over a period in control cohorts of buffalo support our current results. The negative effect of gastrointestinal helminthiasis on milk fat content might be linked with an impairment of nutrient/substance digestions and absorption in the gastrointestinal tract possibly due to the damage caused by the helminths. It could also be associated with reduced appetite during infection as explained by Beriajaya and Copeman [29] for other ruminants.

Our report entails a significant improvement of milk protein after deworming the lactating cows. This finding is also consistent with the study from Nepal [20] and Austria [27]. The results of field clinical trial disclose that deworming using Oxyclozanide and Tetramisole combination (TETROX 3400, DIPSBIOSCIENCE®, India) at the dose rate of 22.6 mg/Kg of body weight in a single dose is effective in the Kucha district, and would help enhance milk yield, fat and protein content. Meanwhile progressive reduction of these values would be anticipated if cows are left untreated.

### Study limitations

In the cross-sectional study design, we demonstrated an insignificant variation in the prevalence of gastrointestinal helminthiasis between dewormed and non-dewormed cows. Although this condition points the possibility of anthelmintic resistance, we could not be certain to generalize this condition is due to resistance without confirmation through anthelmintic efficacy study. Given an Albendazole being the most widely utilized chemical in the district, in our second study design, we confirmed that strategic deworming (use of alternative drug or rarely used drug) has improved milk yield, milk fat and protein. However, the efficacy of these two drugs (Albendazole versus TETROX 3400) should have been compared in terms of parasitological and dairy production parameters.

## Conclusions

In conclusion, this study reveals gastrointestinal helminths are threatening the welfare of lactating cows in Kucha district Ethiopia. A comparable infection status in dewormed and non-dewormed cow point to therapeutic failure possibly due to the development of Albendazole resistance given its extensive utilization in the area. However, an improvement in milk yield, milk fat, and milk protein after deworming using Tetrox® (Tetramisole and Oxyclozanide combination), a drug used by relatively few farmers in the study area, proves the effectiveness of strategic deworming (shifting the most widely utilized to seldom used anthelmintic by the farmers). To better explore the effectiveness of shifting anthelmintic compounds on milk yield, fat, and protein, future studies should include both previous and new drugs in the trial. Moreover, we encourage the study on a field anthelmintic efficacy test in lactating cows in the Kucha district.

## Materials and methods

### Description of study area

**Fig. 5:**
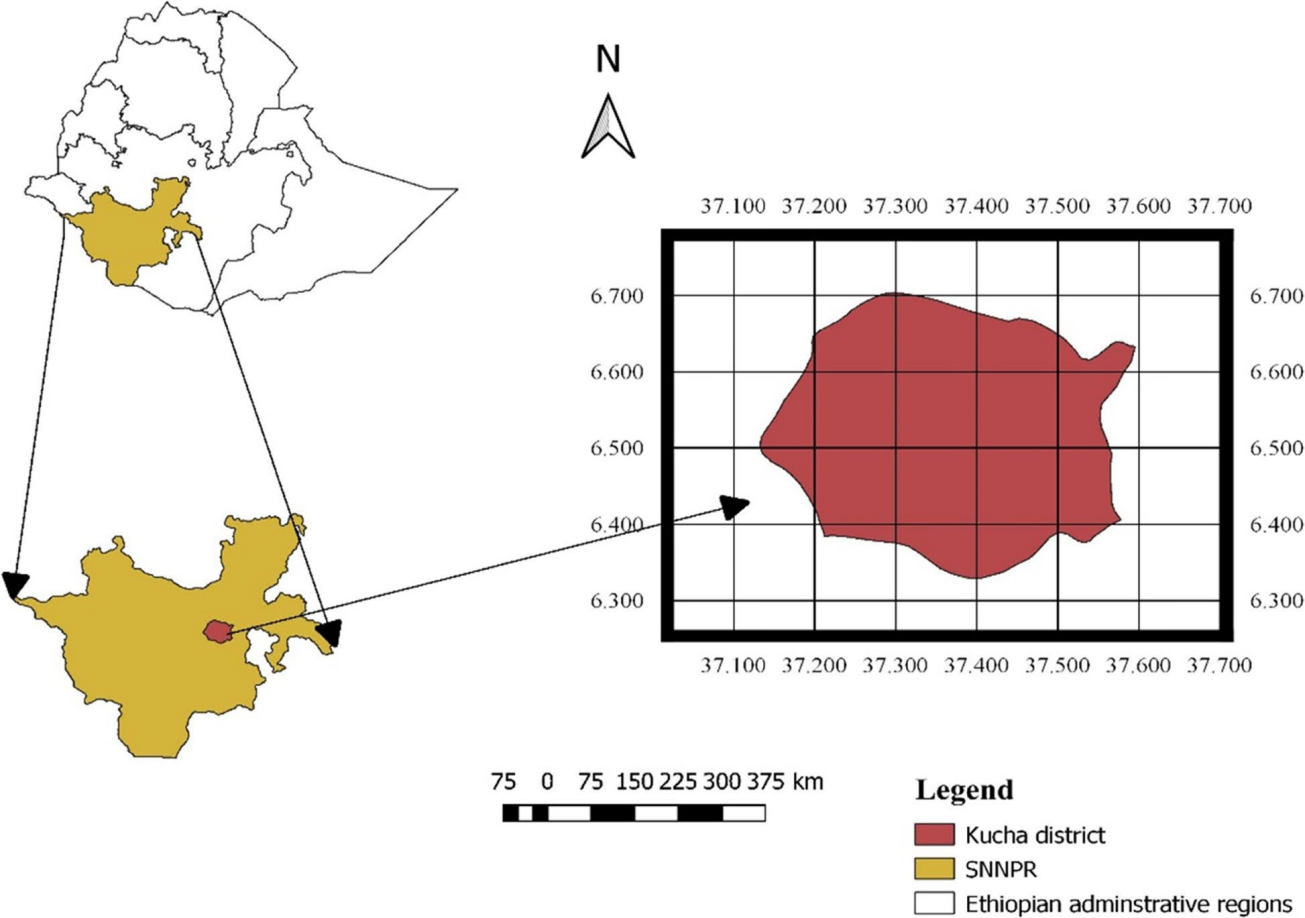
Map of the SNNPR and Kucha district, Ethiopia

Kucha is one of the districts in the Southern Nations, Nationalities, and Peoples' Region (SNNPR) of Ethiopia (Fig. 5). The altitude of the district ranges from 500 to 2700 m above sea level. The mean annual temperature and rainfall respectively range from 17.6 to 27.5 °C and 1401 to 1600 mm. The district has 24 Kebeles (lowest administrative level) with three mixed agro-ecological characteristics based on the traditional agro-ecological classification of the country (Table 3). Dairy products, such as milk and butter are an important source of household income in the district. The farming system of the district is a characteristically mixed livestock-crop type with an extensive and semi-intensive livestock management system.

**Table 3:** Ethiopian traditional agro-ecological zones and their physical characteristics

Traditional name	Agro-ecology	Altitude (m)	Average annual temperature (°C)	Average annual rainfall (mm)
*Kola*	Lowland	500–1500/1800	20–27.5	200–800
*Woinadega*	Midland	1500/1800–2300/2400	17.5/16–20	800–1200
*Dega*	Highland	2300/2400–3200	11.5–17.5/16	1200–2200
*Dega*	Highland	2300/2400–3200	11.5–17.5/16	1200–2200

Source [30]

### Study designs

#### Cross-sectional study

The survey was conducted in the period between January 2021 to March 2021 to execute the aforementioned objectives. The study animals comprised randomly selected lactating cows with different characteristics that were reared under an extensive and semi-intensive management system by smallholder farmers. As recruitment criteria, all lactating cows at different lactation stages, management systems, and physiological states in the Kucha district were selected for enrolling in the study. However, cows under severe illness and those cows whose owner was not willing to give consent for participation were excluded from the study.

The required number of lactating cows for the cross-sectional study was calculated using the formula given below [31], by considering 50% expected prevalence. Accordingly, the sample size required for a cross-sectional study was 384, however, the calculated sample size increased by 10% for contingency purposes; hence final sample size was 422.

*n* = 1.96^2^*P_exp_ (1-P_exp_)/d^2^ Where *n* = sample size, P_exp_ = Expected prevalence, d = desired absolute precision (5%), and 1.96^2^ = *z*-value at 95% confidence level.

A stratified random sampling [31] technique was employed to select lactating cows. In this sampling technique, the district was stratified into highland, midland, and lowland agro-ecology, as there were 24 Kebeles with three distinct agro-ecologies. The number of lactating cows/Kebele was sought from the district's livestock and fisheries office upon official request. Proportional allocation was employed to select both Kebele and cows/Kebele. A schematic sampling procedure was presented in Additional file 2. A single cow/household was selected, as the number of lactating cows for the majority of farmers was few (usually 1–3 cows).

#### Field observation and household interview data collection

Before taking a biological sample, information related to the individual animal, such as breed, age, parity, stage of lactations, pregnancy status, body condition score, and management-related data, including production system, deworming history within the last 4 weeks were obtained through household interview in local language using a structured questionnaire format (Additional file 3) and field observations. The questionnaire was pretested on 5% study population (key informant farmers) one week prior to actual data collection. Animals’ body condition was rated on nine scale (1–9) in which three main conditions, such as poor, medium, and good were intentionally designated by averaging the score [32].

#### Parasitological data collection

Approximately 30 g of faecal sample from the rectum of each cow was taken alongside animal and husbandry information. The samples were placed in a pre-labeled plastic universal screw cap. The samples were placed in the Icebox that had an ice pack to ensure adequate moisture of the faecal sample. All samples were transported right away to Sodo Regional Veterinary Parasitology Laboratory for helminths identification.

In the laboratory, gastrointestinal helminths were identified based on their egg morphology as described in Taylor et al. 2016 [3] following the standard floatation and sedimentation techniques. For faecal floatation, 3 g of sample broken and dissolved in 15 ml of flotation fluid (specfic gravity ~ 1.2). The solution was strained using test seive (250 µm pore size), and the filtrate was poured in to 15 ml plastic test tubes until a reverse meniscus was formed and a coverslip was applied on top of it. The tubes were then allowed to stand for 10 min and finaly the covreslip were removed and mounted on clean microscopic slide for egg identification under light microscope at 100 X total mainification. For sedimenttaion method, the same amount of faecal sample as with in flotation was broken and mixed throughly in 50 ml of water in plastic cup. The mixture was poured in to beaker after straining through 250 µm seive. The filtrate was allowed to sit for one hour. After an hour, supernatants were decanted and more water was added and mixed to repeate sedimentation for the second time. Once supernatant was decanted for the second time, the sediment was mixed up and 0.1% methylene blue was added. Lastly, a drop of mixture was transferred to clean slide to examine eggs under microscope at 100 X maginification power [33].

Cows considered positive if they were shedding at least one type of helminths egg in their faecal material or negative, otherwise.

Quantitative faecal analysis was conducted using the Modified McMaster and Stoll’s techniques with a sensitivity of 50 eggs per gram (EPG) of faeces for positive cows. The McMaster technique was developed at the McMaster laboratory of the University of Sydney and first published in 1939 in the journal of the council for scientific and industrial research by Gordon and Whitlock. Since its first description, many modifications for the technique have been reported. Today, traditional McMaster technique uses two section-counting chamber, which enables to estimate eggs/oocyst from a known volume of fecal suspension by microscopic examination. Briefly, 3 g of faeces was thoroughly mixed in 42 ml of saturated sodim chloride solution (specfic gravity ~ 1.2). The mixture was poured through the kitchen sieve into a laboratory beaker. Aliquates were transferred and filled in to both chambers of McMaster slide uing plastic pasteur pipete. After filling the chambers, the slide was allowed to sit for 5 min. Eggs in both grids of chambers were counted, sumed up and multiplied by 50 to get the number of eggs in a gram of faeces [33].

Stoll’s techniques was applied in order to estimate fluke burden as fluke eggs do not float in sodium chloride solution. A 3 g of feces was emulsified in 42 ml of tap water. The mixture was poured through the kitchen sieve into a laboratory beaker. After that, the solution in the beaker was further strained through a 150 µm laboratory sieve into the conical tube. The tube was allowed to stand for about 3–5 min at room temperature. The supernatant was siphoned off and a drop of 1% methylene blue was added to the sediment. After mixing gently, using a micropipette, 0.15 ml of sediments were transferred to two microscopic glass slides, covered with a cover clip and finally examined under microscope at 100 magnification. The number of egg in both slides were counted, summed up, and multiplied by 50 to get fluke egg per gram (EPG) of faeces as described for McMaster technique [33].

#### Field clinical trial

The study population for field clinical trial comprised lactating cows infected with gastrointestinal helminths based on the first cross-sectional study. The required sample size for field clinical trial was calculated from the hypothesis that the mean milk protein content difference between anthelmintic treated and untreated lactating cows was 0.105% from the previous study [27]. For this calculation, 2-sided directions, *P*-value at 5%, and a study power of 80% were considered by assuming an equal number of animals in the groups. Online epidemiological calculator Epitools (Ausvet®) was used for sample size calculation [34]. Accordingly, 30 cows per group (60 cows in total) were required to participate in the field clinical trial. Lactating cows that met the following inclusion criteria have participated: Criteria; (1) tested positive to at least one type of gastrointestinal helminths, (2) more than 200 EPG of faeces in a previous cross-sectional study (3) early and mid-lactation stage. Cows already dewormed with anthelmintic drugs one month before the study, cow with any overt clinical disease and whose owners were not willing to participate in the study were excluded. Cows enrolled in the study were given an identity code (mark on their horn) to distinguish them from others.

#### Field clinical trial protocol

To take representative positive cows from each agro-ecology, 34, 22, and 4 cows from midland, lowland, and highland respectively were selected using stratified random sampling technique by considering agro-ecology as a stratum and making proportional allocation. All screened cows (*N* = 60) were local Zebu, of which half (*N* = 30) were multiparous at early lactation phase while another half (*N* = 30) were at the second parity and at mid-lactation stages. The body weight of cow was estimated using heart girth measurement. An average body weight of cow was 218.5 kg. Cows in each stratum were randomly assigned into one of the two treatment groups (dewormed and control). Cows in the dewormed group were drenched a bolus that consisted of Oxyclozanide and Tetramisole hydrochloride (TETROX 3400, DIPSBIOSCIENCE®, India) at the dose rate of 22.6 mg/Kg of body weight in a single dose. This anthelmintic was chosen over others due to its relatively low frequency of utilization by the framers in the area (Table 1). Cows in the control groups did not receive any anthelmintic drug throughout the study period.

#### Feeding and cow management

Farmers were instructed to manage their cows indoor until the end of study period. For all cows, the basal diet consisted natural grass hay. Dairy concentrate feed composed of wheat bran; noug seed cake and common salt was given at the rate of 0.5 kg/L of milk every morning on the daily basis. They were also fed locally available vegetable leftover, such as air-dried chips of sweet potato and cassava roots (Manihot esculenta Crants) at the rate of 1 kg/week. Traditional homemade alcoholic product, “*atella*” was offered on weekly basis while water was provided *ad libitum.* Cows were milked twice (early morning and late evening), and allowed to exercise for at least 2 h outside the shelter once in a week.

#### Milk data collection

The volume of milk yield of the individual cow in both treatment groups was recorded five times in a month (at day-0, day-7, day-14, day-21, day-28) using a measuring cylinder during milking. To determine milk protein and fat contents, 10 ml milk sample was collected in a sterile vial on the same days mentioned above. The sample was then, placed in an Ice Box that contained an ice pack for transportation to the dairy technology laboratory of Arba Minch University, College of Agricultural Science. The fat and protein contents of milk collected from each cow were analyzed using an automated Lactoscan milk analyzer ((MILKOTRRONIC®, Bulgaria). The protocol was employed as per the manufacturer's directions.

### Data management and analysis

The information generated from the study was recorded in hard copy format before being prepared in an electronic Microsoft excel sheet. The number of infected cows with at least one type of helminth parasite among total examined cows, the magnitude of each gastrointestinal helminth from a total positive cow, and epidemiological factors were presented in proportion and 95% confidence intervals for proportion using exact binomial test. Mean ± sd was used to present the burden of individual helminths parasites in the positive cow. The association of different hypothesized risk factors, such as agro-ecology, age, parity, lactation period, pregnancy status, deworming history, management system, and breed with overall gastrointestinal helminthiasis (dependent variable) was tested using the binary logistic regression. Odds ratio was calculated to estimate the degree of the associations. P-value and 95% confidence interval for odds ratio were used to judge the statistical significance.

The impact of deworming on milk parameters was analyzed using repeated measure ANOVA in a linear mixed regression model. The models were run using the lmer () function from “lmerTest” package. The models included the main effects (deworming and observation time), their interaction term, and random error (individual animal identity; due to repeated measurement of the same animal). The adjusted average values of milk yield, fat, and protein content were estimated using the function emmeans () from “emmeans” package. Contrast () function was used for post-hock pairwise comparisons in Bonferroni method. The values were presented in the least Squares Means ± Standard Error (LSM ± SE) using a line graph in ggplot () function from “ggplot2” package. For all analysis, 5% marginal error, and 95% confidence level was considered. The R program version 4.1.0. [35] was used to analyze data.

The preliminary analysis showed that the residual error of milk quantity (L), protein (%), and fat (%) content were not normally distributed when assessed using graphically (Q-Q plot) (Additional file 4), which did not allow to use of the parametric test. Hence, data were transformed to log (x-5) scale for the milk yield and square root (x + 5) scale for both fat, and protein content, where x = milk quantity/fat/protein contents, to stabilize variance for parametric analysis. However, the results were presented in back-transformed value for ease of interpretation. The data analysis outputs were presented in Additional file 4.

## Supplementary Information

**Additional file 1: Supplementary Table.** Prevalence of gastrointestinal helminthiasis of lactating cow (*N*=422) and analysis of risk factors using univariable binary logistic regression in Kucha, Ethiopia.

**Additional file 2: Supplementary Figure.** Schematic representation of the sampling procedure of the study subjects in Kucha, Ethiopia.

**Additional file 3:** **Supplementary format.** Data collection format to investigate animal-level and husbandry factors on overall prevalence.

**Additional file 4: Supplementary others.** Milk yield, milk protein and fat contents data analysis outputs using R-program.

## Data Availability

All data generated or analyzed during this study are included in this article and its supplementary information files.

## Abbreviations

EPG: Egg per gram
FEC: Fecal egg count
GIT: Gastrointestinal tract
SNNPR: South Nation Nationality and People Region
