# Supplementary material for: Prevalence and associated factors of gastrointestinal helminthiasis of lactating cow and effect of strategic deworming on milk quantity, fat, and protein in Kucha, Ethiopia
Source: BMC Vet Res. 2022 Apr 25;18:150. doi: 10.1186/s12917-022-03251-2 (PMC9036821; doi:10.1186/s12917-022-03251-2)
Supplement: Supplementary file 1 — Additional file 1: Supplementary Table. Prevalence of gastrointestinal helminthiasis of lactating cow (N=422) and analysis of risk factors using univariable binary logistic regression in Kucha, Ethiopia. [file 12917_2022_3251_MOESM1_ESM.docx]

Additional file 1: Supplementary table on the prevalence (%) of gastrointestinal helminthiasis of lactating cow (N=422) and analysis of risk factors using univariable binary logistic regression in Kucha, Ethiopia

| **Factor** | **No. examined** | **No. infected (%)** | **95% CI (LL-UL)** | **OR** | **95% CI*** | ***P*-value** |
| --- | --- | --- | --- | --- | --- | --- |
| **Agro ecology** |  |  |  |  |  |  |
| Highland | 20 | 10 (50) | (27.2-72.8) | 1.99 | 0.79-5.03 | 0.141 |
| Lowland | 148 | 55 (37.16) | (29.3-45.4) | 1.18 | 0.77-1.79 | 0.453 |
| Midland | 254 | 85 (33.46) | (27.6-39.6) | *Ref* | - | - |
| **Age** |  |  |  |  |  |  |
| Young | 289 | 107 (37.02) | (31.4-42.8) | 1.32 | 0.84-2.11 | 0.233 |
| Old | 16 | 7 (43.75) | (19.7-70.1) | 1.75 | 0.58-5.06 | 0.302 |
| Adult | 117 | 36 (30.76) | (22.5-39.9) | *Ref* | - | - |
| **Average condition score** |  |  |  |  |  |  |
| 2.6 | 252 | 85 (33.73) | (27.9-39.9) | 1.25 | 0.73-2.18 | 0.417 |
| 1.7 | 87 | 41 (47.12) | (36.3-58.1) | 2.19 | 1.17-4.17 | 0.015 |
| 6.3 | 83 | 24 (28.91) | (19.4-39.9) | *Ref* | - | - |
| **Breed** |  |  |  |  |  |  |
| Zebu | 404 | 144 (35.64) | (30.9-40.5) | 1.93 | 0.26-15.18 | 0.517 |
| Zebu x HF cross | 18 | 6 (33.33) | (13.3-59) | *Ref* | - | - |
| **Pregnancy** **status** |  |  |  |  |  |  |
| Pregnant | 102 | 44 (43.13) | (33.3-53.3) | 3.20 | 0.88-15.43 | 0.101 |
| Non-Pregnant | 320 | 106 (33.12) | (27.9-38.5) | *Ref* | - | - |
| **Lactation** **period** |  |  |  |  |  |  |
| Early | 145 | 54 (37.24) | (29.3-45.6) | 1.32 | 0.81-2.16 | 0.260 |
| Late | 116 | 47 (40.51) | (31.5-50) | 0.51 | 0.11-1.85 | 0.343 |
| Mid | 161 | 49 (30.43) | (23.4-38.1) | *Ref* | - | - |
| **Parity** |  |  |  |  |  |  |
| ≥3 | 216 | 81 (37.5) | (31-44.3) | 1.39 | 0.84-2.31 | 0.198 |
| 1 | 79 | 27 (34.17) | (23.8-45.7) | 1.12 | 0.60-2.06 | 0.718 |
| 2 | 127 | 42 (33.07) | (24.9-41.9) | *Ref* | - | - |
| **Management** **system** |  |  |  |  |  |  |
| Semi-intensive | 22 | 8 (36.36) | (17.2-59.3) | 1.62 | 0.26-10.06 | 0.592 |
| Extensive | 400 | 142 (35.5) |  | *Ref* | - | - |
| **Deworming history (before a month)** |  |  |  |  |  |  |
| Non-dewormed | 318 | 119 (37.42) | (32-42.9) | 1.44 | 0.88-2.39 | 0.151 |
| Dewormed | 104 | 31 (29.8) | (21.2-39.5) | *Ref* | - | - |

OR=Odds Ratio, CI=Confidence Interval, LL=lower Limit, UL=Upper Limit, *=Confidence Interval for Odds Ratio, *Ref*=Reference category, HF=Holstein Frisian
