# Supplementary material for: Prevalence and associated factors of gastrointestinal helminthiasis of lactating cow and effect of strategic deworming on milk quantity, fat, and protein in Kucha, Ethiopia
Source: BMC Vet Res. 2022 Apr 25;18:150. doi: 10.1186/s12917-022-03251-2 (PMC9036821; doi:10.1186/s12917-022-03251-2)
Supplement: Supplementary file 2 — Additional file 2: Supplementary Figure. Schematic representation of the sampling procedure of the study subjects in Kucha, Ethiopia. [file 12917_2022_3251_MOESM2_ESM.docx]

Additional file 2: Supplementary figure of schematic representation of the sampling procedure of the study subjects in Kucha, Ethiopia

Kucha District (Total Kebele=24)

N=148 cow recruited

N=254 cow recruited

N=20 cow recruited

No. of Kebele selected =3

(No. of lactating cow=4069)

No. of Kebele selected=3

(No. of lactating cow=7038)

No. of Kebele selected=1

(No. of lactating cow=529)

Highland Kebele (N=3)

Lowland Kebele (N=10)

Midland Kebele (N=11)
