# Supplementary material for: Prevalence and associated factors of gastrointestinal helminthiasis of lactating cow and effect of strategic deworming on milk quantity, fat, and protein in Kucha, Ethiopia
Source: BMC Vet Res. 2022 Apr 25;18:150. doi: 10.1186/s12917-022-03251-2 (PMC9036821; doi:10.1186/s12917-022-03251-2)
Supplement: Supplementary file 3 — Additional file 3: Supplementary format. Data collection format to investigate animal-level and husbandry factors on overall prevalence. [file 12917_2022_3251_MOESM3_ESM.docx]

Additional file 3: Data collection format to investigate animal-level and husbandry factors on overall prevalence

1. **Household information**
   1. Kebele: ________________,
   2. Kebele’s agro ecology: _____________,
   3. Owner/head of household: M/F
2. **Animal level information**
   1. Dairy cow herd size: ______
   2. Number of lactating cow: ______
   3. Breed of selected lactating dairy cows for study: a) local b) crossbred c) exotic
   4. Age: a) young (1-6 years) b) adult (7-10 years) c) old (>10 years)
   5. Lactation stage of selected cow: a) early (birth-3months) b) mid (3-6months) c) late (above 6 months)
   6. Pregnancy status: a) pregnant b) non-pregnant
   7. Parity: a) 1 b) 2 c) 3 d) >3
   8. Management system: a) extensive b) intensive c) semi-intensive
   9. Deworming status with in the past 4 weeks: a) dewormed b) not dewormed
   10. If your answer to the question indicated above (2.6) is a, which compound have you used? a) Albendazole b) Ivermectin c) Tetramizole d) Tetrox
   11. Presence of other livestock in the household: a) yes b) no
