# Supplementary material for: Prevalence and associated factors of gastrointestinal helminthiasis of lactating cow and effect of strategic deworming on milk quantity, fat, and protein in Kucha, Ethiopia
Source: BMC Vet Res. 2022 Apr 25;18:150. doi: 10.1186/s12917-022-03251-2 (PMC9036821; doi:10.1186/s12917-022-03251-2)
Supplement: Supplementary file 4 — Additional file 4: Supplementary others. Milk yield, milk protein and fat contents data analysis outputs using R-program. [file 12917_2022_3251_MOESM4_ESM.docx]

Additional file 4: Analysis output of milk yield, milk protein and fat contents data analysis outputs using R-program.

**Effect of deworming on fat content**

Check of Normality

**
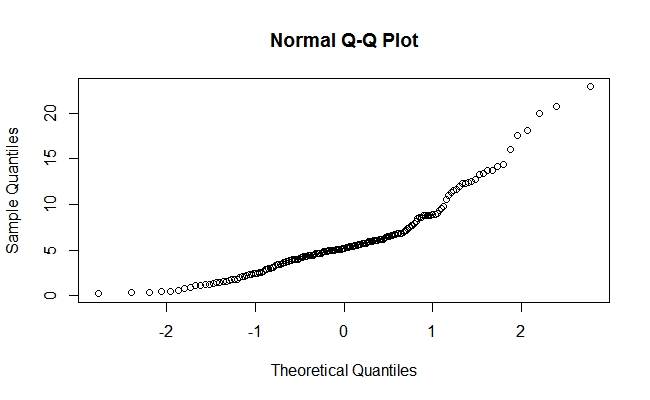

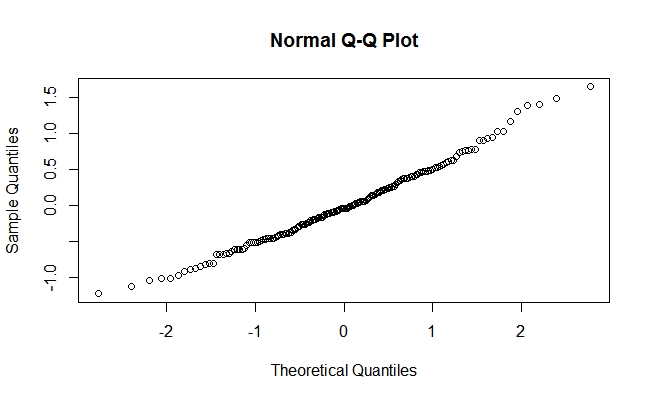
**

1. Before transformation b) After transformation

library(lmerTest)

Model<- lmer(sqrt(`fat(%)`+0.5)~treatment*day+(1|ID),data = Dat) #### transformed

summary (Model) ##### to see the association of fixed effects, interactions on the outcome

library(emmeans)

EMM<-emmeans(Model,specs = pairwise~treatment:day,type="response") ### provides LSM with SE in back transformed values as shown below

**1. Least Squares Means and Standard Errors for various sample collection period (back transformed values)**

> mean$emmeans

treatment day response SE df lower.CL upper.CL

dewormed day-0 5.06 0.417 165 4.27 5.92

control day-0 5.75 0.444 165 4.91 6.66

dewormed day-7 5.07 0.417 165 4.28 5.93

control day-7 5.86 0.448 165 5.00 6.77

dewormed day-14 6.39 0.468 165 5.50 7.35

control day-14 3.96 0.369 165 3.26 4.72

dewormed day-21 6.39 0.469 165 5.50 7.35

control day-21 3.83 0.363 165 3.14 4.58

dewormed day-28 6.41 0.469 165 5.51 7.37

control day-28 3.83 0.362 165 3.14 4.57

Degrees-of-freedom method: kenward-roger

Confidence level used: 0.95

Intervals are back-transformed from the sqrt scale

> mean$contrasts

contrast estimate SE df t.ratio p.value

(dewormed day-0) - (control day-0) -0.148057 0.1310 165 -1.130 0.9809

(dewormed day-0) - (dewormed day-7) -0.001322 0.0985 232 -0.013 1.0000

(dewormed day-0) - (control day-7) -0.169302 0.1310 165 -1.292 0.9542

(dewormed day-0) - (dewormed day-14) -0.277307 0.0985 232 -2.814 0.1378

(dewormed day-0) - (control day-14) 0.260937 0.1310 165 1.991 0.6064

(dewormed day-0) - (dewormed day-21) -0.278256 0.0985 232 -2.824 0.1347

(dewormed day-0) - (control day-21) 0.294069 0.1310 165 2.244 0.4313

(dewormed day-0) - (dewormed day-28) -0.280935 0.0985 232 -2.851 0.1260

(dewormed day-0) - (control day-28) 0.294638 0.1310 165 2.248 0.4284

(control day-0) - (dewormed day-7) 0.146736 0.1310 165 1.120 0.9821

(control day-0) - (control day-7) -0.021244 0.0985 232 -0.216 1.0000

(control day-0) - (dewormed day-14) -0.129249 0.1310 165 -0.986 0.9927

(control day-0) - (control day-14) 0.408994 0.0985 232 4.151 0.0019

(control day-0) - (dewormed day-21) -0.130198 0.1310 165 -0.994 0.9923

(control day-0) - (control day-21) 0.442127 0.0985 232 4.487 0.0005

(control day-0) - (dewormed day-28) -0.132878 0.1310 165 -1.014 0.9911

(control day-0) - (control day-28) 0.442696 0.0985 232 4.493 0.0005

(dewormed day-7) - (control day-7) -0.167980 0.1310 165 -1.282 0.9564

(dewormed day-7) - (dewormed day-14) -0.275985 0.0985 232 -2.801 0.1423

(dewormed day-7) - (control day-14) 0.262258 0.1310 165 2.001 0.5993

(dewormed day-7) - (dewormed day-21) -0.276934 0.0985 232 -2.810 0.1391

(dewormed day-7) - (control day-21) 0.295391 0.1310 165 2.254 0.4246

(dewormed day-7) - (dewormed day-28) -0.279614 0.0985 232 -2.838 0.1302

(dewormed day-7) - (control day-28) 0.295960 0.1310 165 2.259 0.4217

(control day-7) - (dewormed day-14) -0.108005 0.1310 165 -0.824 0.9981

(control day-7) - (control day-14) 0.430238 0.0985 232 4.366 0.0008

(control day-7) - (dewormed day-21) -0.108954 0.1310 165 -0.831 0.9980

(control day-7) - (control day-21) 0.463371 0.0985 232 4.702 0.0002

(control day-7) - (dewormed day-28) -0.111634 0.1310 165 -0.852 0.9976

(control day-7) - (control day-28) 0.463940 0.0985 232 4.708 0.0002

(dewormed day-14) - (control day-14) 0.538243 0.1310 165 4.107 0.0025

(dewormed day-14) - (dewormed day-21) -0.000949 0.0985 232 -0.010 1.0000

(dewormed day-14) - (control day-21) 0.571376 0.1310 165 4.360 0.0009

(dewormed day-14) - (dewormed day-28) -0.003629 0.0985 232 -0.037 1.0000

(dewormed day-14) - (control day-28) 0.571945 0.1310 165 4.365 0.0009

(control day-14) - (dewormed day-21) -0.539193 0.1310 165 -4.115 0.0024

(control day-14) - (control day-21) 0.033133 0.0985 232 0.336 1.0000

(control day-14) - (dewormed day-28) -0.541872 0.1310 165 -4.135 0.0022

(control day-14) - (control day-28) 0.033702 0.0985 232 0.342 1.0000

(dewormed day-21) - (control day-21) 0.572325 0.1310 165 4.368 0.0009

(dewormed day-21) - (dewormed day-28) -0.002680 0.0985 232 -0.027 1.0000

(dewormed day-21) - (control day-28) 0.572894 0.1310 165 4.372 0.0009

(control day-21) - (dewormed day-28) -0.575005 0.1310 165 -4.388 0.0008

(control day-21) - (control day-28) 0.000569 0.0985 232 0.006 1.0000

(dewormed day-28) - (control day-28) 0.575574 0.1310 165 4.392 0.0008

Note: contrasts are still on the sqrt scale

Degrees-of-freedom method: kenward-roger

P value adjustment: tukey method for comparing a family of 10 estimates

**Effect of deworming on Milk yield**

Check of Normality


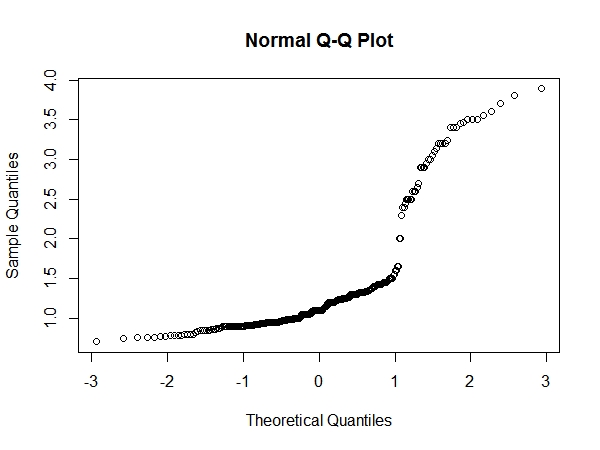

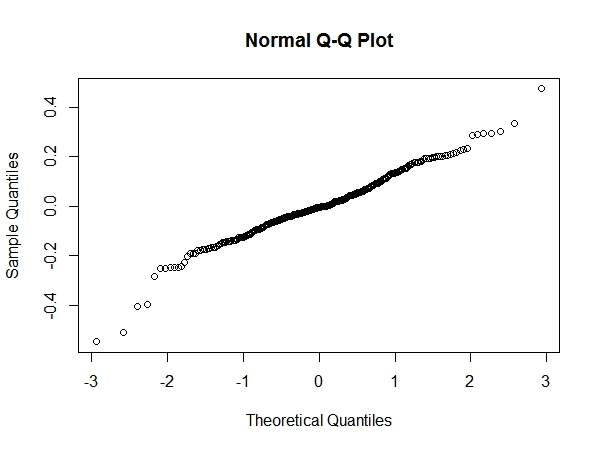


Figure 2: Before transformation (left) and after transformation (right) of milk yield data

library(lmerTest)

Model<-lmer(log(milk-0.5)~treatment*day+(1|ID),data = data)

summary(Model)

library(emmeans)

EMM<-emmeans(Model,specs = pairwise~treatment:day,type="response")

EMM$emmeans

EMM$contrasts

**1. LSM±SE (Milk yield)**

> EMM$emmeans

treatment day response SE df lower.CL upper.CL

dewormed 0 0.558 0.0589 64.7 0.452 0.689

control 0 0.461 0.0487 64.7 0.373 0.569

dewormed 7 0.701 0.0740 64.7 0.568 0.865

control 7 0.570 0.0601 64.7 0.461 0.703

dewormed 14 0.919 0.0970 64.7 0.744 1.135

control 14 0.610 0.0644 64.7 0.494 0.753

dewormed 21 1.036 0.1094 64.7 0.839 1.280

control 21 0.657 0.0694 64.7 0.532 0.811

dewormed 28 1.007 0.1063 64.7 0.815 1.243

control 28 0.622 0.0657 64.7 0.504 0.768

Degrees-of-freedom method: kenward-roger

Confidence level used: 0.95

Intervals are back-transformed from the log scale

**2. Pairwise comparison (post-hock)**

**> EMM$contrasts**

contrast ratio SE df null t.ratio p.value

dewormed 0 / control 0 1.210 0.1807 64.7 1 1.278 0.9551

dewormed 0 / dewormed 7 0.796 0.0308 232.0 1 -5.900 <.0001

dewormed 0 / control 7 0.979 0.1462 64.7 1 -0.139 1.0000

dewormed 0 / dewormed 14 0.607 0.0235 232.0 1 -12.908 <.0001

dewormed 0 / control 14 0.915 0.1366 64.7 1 -0.595 0.9998

dewormed 0 / dewormed 21 0.538 0.0208 232.0 1 -16.018 <.0001

dewormed 0 / control 21 0.849 0.1268 64.7 1 -1.094 0.9836

dewormed 0 / dewormed 28 0.554 0.0214 232.0 1 -15.263 <.0001

dewormed 0 / control 28 0.897 0.1339 64.7 1 -0.729 0.9992

control 0 / dewormed 7 0.658 0.0982 64.7 1 -2.806 0.1564

control 0 / control 7 0.809 0.0313 232.0 1 -5.470 <.0001

control 0 / dewormed 14 0.502 0.0749 64.7 1 -4.620 0.0007

control 0 / control 14 0.756 0.0292 232.0 1 -7.231 <.0001

control 0 / dewormed 21 0.445 0.0664 64.7 1 -5.426 <.0001

control 0 / control 21 0.702 0.0271 232.0 1 -9.160 <.0001

control 0 / dewormed 28 0.458 0.0684 64.7 1 -5.230 0.0001

control 0 / control 28 0.741 0.0287 232.0 1 -7.751 <.0001

dewormed 7 / control 7 1.230 0.1837 64.7 1 1.389 0.9262

dewormed 7 / dewormed 14 0.763 0.0295 232.0 1 -7.007 <.0001

dewormed 7 / control 14 1.149 0.1716 64.7 1 0.933 0.9947

dewormed 7 / dewormed 21 0.676 0.0261 232.0 1 -10.118 <.0001

dewormed 7 / control 21 1.067 0.1593 64.7 1 0.434 1.0000

dewormed 7 / dewormed 28 0.696 0.0269 232.0 1 -9.363 <.0001

dewormed 7 / control 28 1.127 0.1682 64.7 1 0.799 0.9984

control 7 / dewormed 14 0.620 0.0925 64.7 1 -3.204 0.0605

control 7 / control 14 0.934 0.0361 232.0 1 -1.761 0.7590

control 7 / dewormed 21 0.550 0.0821 64.7 1 -4.009 0.0058

control 7 / control 21 0.867 0.0335 232.0 1 -3.690 0.0103

control 7 / dewormed 28 0.566 0.0845 64.7 1 -3.814 0.0107

control 7 / control 28 0.916 0.0354 232.0 1 -2.280 0.4059

dewormed 14 / control 14 1.507 0.2250 64.7 1 2.748 0.1771

dewormed 14 / dewormed 21 0.887 0.0343 232.0 1 -3.110 0.0636

dewormed 14 / control 21 1.399 0.2088 64.7 1 2.248 0.4359

dewormed 14 / dewormed 28 0.913 0.0353 232.0 1 -2.355 0.3579

dewormed 14 / control 28 1.477 0.2205 64.7 1 2.613 0.2328

control 14 / dewormed 21 0.588 0.0878 64.7 1 -3.553 0.0232

control 14 / control 21 0.928 0.0359 232.0 1 -1.929 0.6492

control 14 / dewormed 28 0.606 0.0904 64.7 1 -3.358 0.0402

control 14 / control 28 0.980 0.0379 232.0 1 -0.520 1.0000

dewormed 21 / control 21 1.578 0.2355 64.7 1 3.054 0.0882

dewormed 21 / dewormed 28 1.030 0.0398 232.0 1 0.755 0.9991

dewormed 21 / control 28 1.666 0.2487 64.7 1 3.419 0.0340

control 21 / dewormed 28 0.653 0.0974 64.7 1 -2.858 0.1393

control 21 / control 28 1.056 0.0408 232.0 1 1.410 0.9233

dewormed 28 / control 28 1.618 0.2415 64.7 1 3.223 0.0491

Degrees-of-freedom method: kenward-roger

P value adjustment: tukey method for comparing a family of 10 estimates

Tests are performed on the log scale

**Effect of deworming on protein**

Normality check


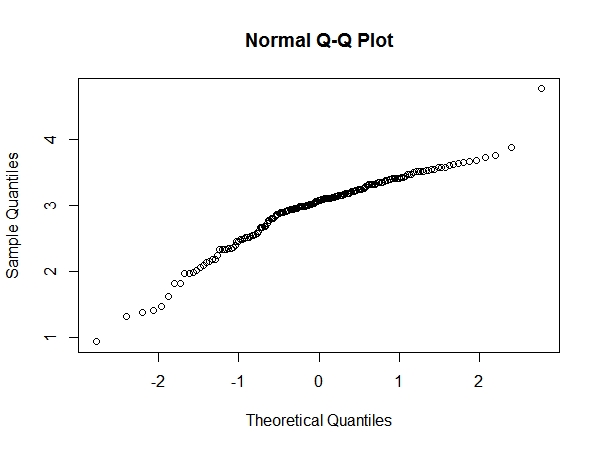

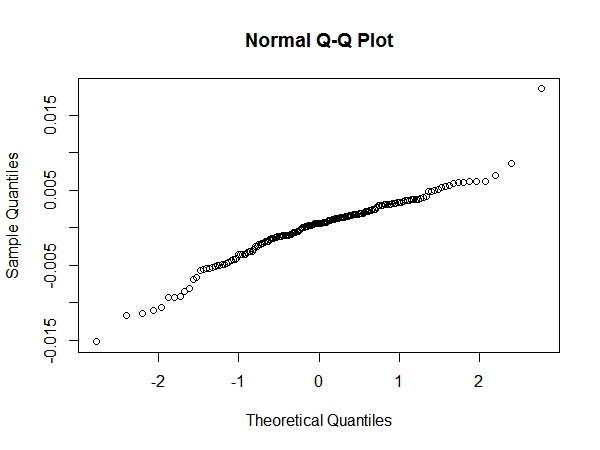


Figure 3: Distribution of untransformed (left) and transformed residuals (right) of milk protein

model<-lmer(sqrt(`prot(%)`+0.5)~treatment*day+(1|ID),data = Dat)

summary(model)

library(emmeans)

Yield

EMM<-emmeans(model,specs = pairwise~treatment:day,type="response")

EMM$emmeans

treatment day response SE df lower.CL upper.CL

dewormed day-0 3.17 0.0731 237 3.03 3.32

control day-0 3.43 0.0760 237 3.28 3.58

dewormed day-7 3.53 0.0772 237 3.38 3.69

control day-7 3.28 0.0743 237 3.13 3.43

dewormed day-14 3.82 0.0802 237 3.66 3.98

control day-14 3.53 0.0771 237 3.38 3.68

dewormed day-21 3.83 0.0803 237 3.67 3.99

control day-21 3.53 0.0771 237 3.38 3.68

dewormed day-28 3.83 0.0803 237 3.67 3.99

control day-28 3.53 0.0771 237 3.38 3.68

Degrees-of-freedom method: kenward-roger

Confidence level used: 0.95

Intervals are back-transformed from the sqrt scale

> EMM$contrasts

contrast estimate SE df t.ratio p.value

(dewormed day-0) - (control day-0) -7.19e-02 0.0290 237 -2.478 0.2858

(dewormed day-0) - (dewormed day-7) -9.98e-02 0.0253 232 -3.937 0.0042

(dewormed day-0) - (control day-7) -3.07e-02 0.0290 237 -1.057 0.9882

(dewormed day-0) - (dewormed day-14) -1.74e-01 0.0253 232 -6.869 <.0001

(dewormed day-0) - (control day-14) -9.80e-02 0.0290 237 -3.378 0.0287

(dewormed day-0) - (dewormed day-21) -1.76e-01 0.0253 232 -6.933 <.0001

(dewormed day-0) - (control day-21) -9.80e-02 0.0290 237 -3.378 0.0287

(dewormed day-0) - (dewormed day-28) -1.77e-01 0.0253 232 -6.971 <.0001

(dewormed day-0) - (control day-28) -9.82e-02 0.0290 237 -3.385 0.0281

(control day-0) - (dewormed day-7) -2.79e-02 0.0290 237 -0.961 0.9941

(control day-0) - (control day-7) 4.12e-02 0.0253 232 1.627 0.8337

(control day-0) - (dewormed day-14) -1.02e-01 0.0290 237 -3.522 0.0181

(control day-0) - (control day-14) -2.61e-02 0.0253 232 -1.030 0.9901

(control day-0) - (dewormed day-21) -1.04e-01 0.0290 237 -3.578 0.0150

(control day-0) - (control day-21) -2.61e-02 0.0253 232 -1.030 0.9901

(control day-0) - (dewormed day-28) -1.05e-01 0.0290 237 -3.611 0.0134

(control day-0) - (control day-28) -2.63e-02 0.0253 232 -1.038 0.9896

(dewormed day-7) - (control day-7) 6.91e-02 0.0290 237 2.382 0.3416

(dewormed day-7) - (dewormed day-14) -7.43e-02 0.0253 232 -2.932 0.1028

(dewormed day-7) - (control day-14) 1.75e-03 0.0290 237 0.060 1.0000

(dewormed day-7) - (dewormed day-21) -7.59e-02 0.0253 232 -2.996 0.0869

(dewormed day-7) - (control day-21) 1.76e-03 0.0290 237 0.061 1.0000

(dewormed day-7) - (dewormed day-28) -7.69e-02 0.0253 232 -3.034 0.0785

(dewormed day-7) - (control day-28) 1.55e-03 0.0290 237 0.054 1.0000

(control day-7) - (dewormed day-14) -1.43e-01 0.0290 237 -4.943 0.0001

(control day-7) - (control day-14) -6.74e-02 0.0253 232 -2.657 0.1977

(control day-7) - (dewormed day-21) -1.45e-01 0.0290 237 -4.999 <.0001

(control day-7) - (control day-21) -6.74e-02 0.0253 232 -2.657 0.1977

(control day-7) - (dewormed day-28) -1.46e-01 0.0290 237 -5.032 <.0001

(control day-7) - (control day-28) -6.76e-02 0.0253 232 -2.665 0.1943

(dewormed day-14) - (control day-14) 7.61e-02 0.0290 237 2.622 0.2135

(dewormed day-14) - (dewormed day-21) -1.62e-03 0.0253 232 -0.064 1.0000

(dewormed day-14) - (control day-21) 7.61e-02 0.0290 237 2.622 0.2134

(dewormed day-14) - (dewormed day-28) -2.58e-03 0.0253 232 -0.102 1.0000

(dewormed day-14) - (control day-28) 7.59e-02 0.0290 237 2.615 0.2166

(control day-14) - (dewormed day-21) -7.77e-02 0.0290 237 -2.677 0.1890

(control day-14) - (control day-21) 4.20e-06 0.0253 232 0.000 1.0000

(control day-14) - (dewormed day-28) -7.87e-02 0.0290 237 -2.711 0.1755

(control day-14) - (control day-28) -1.97e-04 0.0253 232 -0.008 1.0000

(dewormed day-21) - (control day-21) 7.77e-02 0.0290 237 2.678 0.1890

(dewormed day-21) - (dewormed day-28) -9.63e-04 0.0253 232 -0.038 1.0000

(dewormed day-21) - (control day-28) 7.75e-02 0.0290 237 2.671 0.1919

(control day-21) - (dewormed day-28) -7.87e-02 0.0290 237 -2.711 0.1754

(control day-21) - (control day-28) -2.01e-04 0.0253 232 -0.008 1.0000

(dewormed day-28) - (control day-28) 7.85e-02 0.0290 237 2.704 0.1782

Note: contrasts are still on the sqrt scale

Degrees-of-freedom method: kenward-roger

P value adjustment: tukey method for comparing a family of 10 estimates
